# Using the Technology Acceptance Model to explore community dwelling older adults’ perceptions of a 3D interior design application to facilitate pre-discharge home adaptations

**DOI:** 10.1186/s12911-015-0190-2

**Published:** 2015-08-26

**Authors:** Arthur G. Money, Anita Atwal, Katherine L. Young, Yasmin Day, Lesley Wilson, Kevin G. Money

**Affiliations:** 1 Department of Computer Science, Brunel University, Uxbridge, UB8 3PH UK; 2 Department of Clinical Sciences, Brunel University, Uxbridge, UB8 3PH UK; 3 Henley Business School, Greenlands, Henley-on-Thames, RG9 3AU UK

**Keywords:** Occupational therapy, Pre-discharge home visits, Virtual reality, 3D, Collaboration, Engagement, Empowerment, Technology assisted care, Patient perceptions

## Abstract

**Background:**

In the UK occupational therapy pre-discharge home visits are routinely carried out as a means of facilitating safe transfer from the hospital to home. Whilst they are an integral part of practice, there is little evidence to demonstrate they have a positive outcome on the discharge process. Current issues for patients are around the speed of home visits and the lack of shared decision making in the process, resulting in less than 50 % of the specialist equipment installed actually being used by patients on follow-up. To improve practice there is an urgent need to examine other ways of conducting home visits to facilitate safe discharge. We believe that Computerised 3D Interior Design Applications (CIDAs) could be a means to support more efficient, effective and collaborative practice. A previous study explored practitioners perceptions of using CIDAs; however it is important to ascertain older adult’s views about the usability of technology and to compare findings. This study explores the perceptions of community dwelling older adults with regards to adopting and using CIDAs as an assistive tool for the home adaptations process.

**Methods:**

Ten community dwelling older adults participated in individual interactive task-focused usability sessions with a customised CIDA, utilising the think-aloud protocol and individual semi-structured interviews. Template analysis was used to carry out both deductive and inductive analysis of the think-aloud and interview data. Initially, a deductive stance was adopted, using the three pre-determined high-level themes of the technology acceptance model (TAM): Perceived Usefulness (PU), Perceived Ease of Use (PEOU), Actual Use (AU). Inductive template analysis was then carried out on the data within these themes, from which a number of sub-thmes emerged.

**Results:**

Regarding PU, participants believed CIDAs served as a useful visual tool and saw clear potential to facilitate shared understanding and partnership in care delivery. For PEOU, participants were able to create 3D home environments however a number of usability issues must still be addressed. The AU theme revealed the most likely usage scenario would be collaborative involving both patient and practitioner, as many participants did not feel confident or see sufficient value in using the application autonomously.

**Conclusions:**

This research found that older adults perceived that CIDAs were likely to serve as a valuable tool which facilitates and enhances levels of patient/practitioner collaboration and empowerment. Older adults also suggested a redesign of the interface so that less sophisticated dexterity and motor functions are required. However, older adults were not confident, or did not see sufficient value in using the application autonomously. Future research is needed to further customise the CIDA software, in line with the outcomes of this study, and to explore the potential of collaborative application patient/practitioner-based deployment.

## Background

It is becoming increasingly accepted that provision of good quality care is synonymous with the notion of providing more person-centred care, and increasing the levels of patient involvement in the decisions that are made about their care [1, 2]. The world population is ageing, one-in-six of the UK’s population is currently aged 65 and over, which by 2050 will have risen to one-in-four [3]. In addition to the goal of providing better quality care, the increased burden that an ageing population puts on health care services is driving a search for new technology-enhanced modes of care delivery that will be capable of meeting patient needs whilst also adhering to anticipated budgetary constraints [4]. The development and use of new software applications and information and communication technologies is one of the few areas that has the potential to reduce costs whilst improving the quality of care and facilitating the provision of patient-centred care [5]. In particular, developing information and communication technology applications that enable patients to participate alongside practitioners when making care decisions is a key UK government strategy which responds to the challenge of catering for the increasing health care demands of an ageing population [6].

The World Health Organisation’s International Classification of Functioning, Disability and Health (ICF) framework [7] highlights that loss of independence is not only linked to body function but also to environmental factors. Environmental factors interact with a health condition to either create a disability or restore functioning, depending on whether the environmental factor can be regarded as a barrier or a facilitator [8]. The pre-discharge home visit is an integral part of the discharge process and involves taking older adults to their home for a short period of time to assess their ability to perform some occupations of daily living within their own environment [9]. Home modifications and the installation of assistive equipment can impact on a client’s life by better supporting safe independent living, but these changes are not always viewed in a positive way by patients. By seeking out ways to include clients in decision making, Occupational Therapists have the opportunity to reinforce their desire to be person-centred. However existing research has found dissatisfaction with aspects of the occupational therapy home visit, despite there being evidence that there is a consistent positive association between patient experience and clinical outcomes [10]. Some older adults found the home visit experience demoralising, daunting, and anxiety-provoking because of weak communication, poor preparation, and their lack of involvement in decision making [11]. As a consequence, less than 50 % of assistive equipment installed in homes, as a result carrying out pre-discharge home visits, is actually used by patients [12]. This low level of engagement may not be surprising, when considering how personalised and sensitive the home environment is considered to be by patients.

Occupational Therapists recognise that the relationship between a client and their home can have many layers relating to identity, security, and personal history [13]. They also recognise that clients are the best source of information regarding how day-to-day occupations are carried out at home. However, there are no existing tools or techniques specifically designed to support the collaborative process, which should occur between the patient and practitioner, to visualise, negotiate, and make decisions about how the home environment may be altered/adapted to best suit and facilitate patient needs [14]. There is an urgent need to address the high levels of equipment abandonment that occur as a result of the pre-discharge home visit process [12]. One approach to addressing this need is to explore how technology, particularly 3D interior design software applications, may be used to help patients and practitioners collaborate effectively during the process. Moreover, there is a need to develop tools which enable patients to communicate the importance of the intricacies of their personal home space to practitioners, and for practitioners to better visually simulate and communicate the options that are available to patients with regards to home adaptations. Likewise there is a need to enable patients to communicate how proposed adaptations are perceived to impact upon their personal home environment.

### Computerised 3D design applications for home adaptation

A computerised three-dimensional virtual environment (3DVE) is defined as an environment which “capitalizes upon natural aspects of human perception by extending visual information in three spatial dimensions” and “enables the user to interact with the displayed data” [15]. The areas in which 3DVEs have been applied span across a range of domains which include interior design [16], health and wellbeing [17], military and defence [18, 19], education [20] and gaming for health [21]. Furthermore, mobile 3DVE applications are becoming recognised as valuable tools that may be applied to a range of healthcare scenarios [22]. More specifically within the domain of home interior design, computerised 3D interior design applications (CIDA) are one form of 3DVE which serve as a valuable assistive 3D visualisation tool for negotiating adaptations between designers and home owners [23]. CIDA supports the process of exploring a variety of potential interior designs and home adaptations, and enables users to weigh up the relative benefits and challenges which these variations present before committing to having the adaptations made in reality. There are a number of potential benefits that CIDA could bring to the home adaptation process, such as improving the extent to which the practitioner and patient are able to negotiate, collaborate and understand the range of adaptation options available. Furthermore, by simulating potential adaptations using CIDA, misconceptions and potential misunderstandings of proposed changes may be brought to the forefront of a discussion, hence enabling better engagement between client and practitioner and facilitating the process of consensus building [24]. CIDA, therefore, has the potential to serve as a tool that enables patients and practitioners to present simulated representations of the patient’s home and to jointly discuss the interior layout of the home and explore the range of adaptations and specialist equipment that may potentially be placed within it. This would not only provide valuable opportunities to engage in shared decision making, but would also opportunity for practitioners to develop a more detailed understanding of their patients and for patients to better understand the function, and potential benefits, of items of specialist assistive equipment. Effective use of CIDA has the potential of empowering patients to participate as more equal partners in decision making, hence potentially averting the daunting, demoralising and anxiety provoking experience that traditional practitioner-led home-based visits are so often found to be by some patients [9].

### CIDA research relating to home adaptations

Despite the potential benefits, there are many challenges to realising the utilisation of CIDA in practice. Particularly as occupational therapists have been found to be reluctant to use information and communication technology in practice [25]. Previous occupational therapist focused research related to the current study, exploring the concept of utilising CIDA in practice, found that some therapists believed CIDA should be treated with caution so as not to be seen as a tool that would replace the role of the occupational therapist. However, they also viewed it as a tool that could enhance their status within the health care profession and improve communication [14]. In a later study, trialling a more developed version of a CIDA software application, occupational therapists reported that they were able to use the software. However, some ‘fine tuning’ was needed, such as improving the look and feel of the application and expanding the library of household items that may be included in models of patients’ homes, if the application is to be optimally used in practice [26]. Whilst the views of therapists are important, patient-focused research has shown that they are more likely to adopt technologies if these are viewed as usable and are perceived to be compatible with their needs [27]. In particular, it is important to ascertain older adults’ views of technology and its potential applicability in practice. A systematic review and critical evaluation of smart technologies and their potential use in enhancing social connectedness revealed that some technologies augment the beneficial effects of traditional older adult care practices [28]. Furthermore, another systematic review found that older adults readily accepted smart-home technologies, providing they physical activity, function and independence [29]. Other studies relating specifically to older adults and technology acceptance have also found that perceived usefulness of technology, its ease of use, and effort expectancy are key factors that impact on adoption of new technologies [30, 31]. Other older adult patient focused studies have found that unhelpful features, inconsistency in interface design, and concerns relating to the reliability and stability of the application are features which are seen in a particularly negative light by this cohort [32].

The application of CIDA must be accepted by patients if it is to serve as a feasible tool which may be used by patients within occupational therapy practice [33]. If CIDA are not perceived as usable or likable in the eyes of the patient, it is unlikely that the technology will remain in use long enough for an evidence-base to be explored and established. Although previous studies have explored the use of CIDA from the occupational therapists’ perspective [27, 33], no existing research explores patient perceptions of utilising CIDA within the pre-discharge home visit process. It is therefore crucial that patient perceptions of CIDA technology is explored, given the potential impact patient perceptions of such applications could have on the potential long term integration of such technology in practice.

### Patient attitudes towards technology

Involving the patient at every stage of the health care technology design, development and deployment process is crucial if new technologies are to be optimally used and accepted in practice [34]. Existing research suggests that patients are more likely to engage with technologies if they are usable and are perceived to be compatible with patient needs [27, 35]. If CIDA is to be adopted in practice and serve as a useful tool, it must be perceived as being easy to use, useful and patients must see it as having utility in practice [33]. Gaining insights into patient acceptance of new technologies has been the subject of much research in recent years, in particular, much effort has been invested into understanding users’ reactions and motivations to using health care technology in practice [36, 37]. Perhaps the most widely used theory to evaluate user attitudes towards the acceptance of technology is the technology acceptance model (TAM) [38]. Although it is a relatively straightforward model, the key TAM constructs have been seen to typically explain more than forty percent of user related issues around technology acceptance [36].

The model suggests that users’ behavioural intention to use and their Actual Use (AU) of technology are mediated by two factors: Perceived Usefulness (PU): ‘the extent to which the user perceives that the new technology will aid them in performing the task at hand’, and Perceived Ease of Use (PEoU): ‘the extent to which the individual believes using the technology would be free of effort’ [39]. TAM is now increasingly being applied within the healthcare research domain [40]. Although, a recent systematic review that examined acceptance of technology for aging in place found that post-implementation research on technology acceptance by community-dwelling older adults is scarce [41]. Interestingly Or and Karsh [42], in a systematic review of acceptance of consumer health information technology, found that no studies examined the impact of social and task factors on acceptance and few tested the effects of organisational or environmental factors on acceptance. In recent years, the TAM factors have been increasingly used as part of more qualitative interview studies to structure conversation around the key themes that have been found to be key in the user technology acceptance [43].

This study builds on a previous study, which focused on occupational therapists’ perceptions of CIDA [26], to gain insights into patient perspectives of using CIDA as a tool to aid the pre-discharge home visits process. Therefore, the aim of this study is to explore community dwelling older adults’ perceptions of using CIDA in terms of its perceived usefulness, ease of use, and actual use and to consider the potential barriers and opportunities of using a CIDA application as an assistive tool within the pre-discharge home visits process.

## Methods

**Fig. 1:**
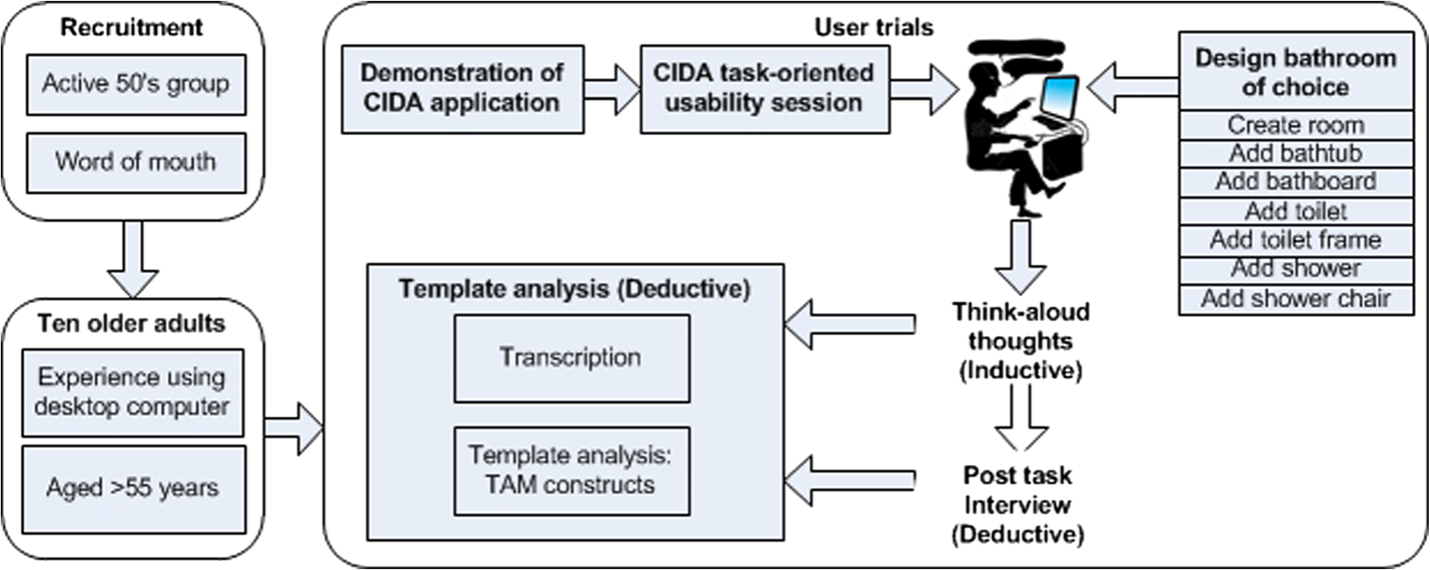
Overview of trials methods and process

This section provides details of the methods used for user trials, data collection and analysis. Figure 1 provides an overview of the methods used and process followed.

### Participants

The study sample focused on recruitment of community-dwelling older adults for participation within the task-oriented usability sessions. Convenience sampling was used for recruitment of participants to this study, for which a total of 10 participants were recruited. The inclusion criteria were that participants were aged over 55, had basic computer literacy skills, i.e., they were familiar with using desktop computers, and considered themselves to be active and healthy. With regards to the computer literacy inclusion criterion, this was fulfilled by participants, who all self-reported that they were familiar with carrying out everyday computer tasks such as using a desktop or laptop computer to check email and browse internet content. Consequently, no usability issues were observed during the trials with regards to participants manipulating the input/output hardware devices (i.e., the desktop, mouse and keyboard setup). The rationale behind recruiting a sample of participants who possessed basic computer literacy skills was in part motivated by the aim of this study, which was to gain insights into older adults’ perceptions specifically of the CIDA software and its functionality. Recruiting participants with basic computer literacy skills helped to ensure that the focus remained on the CIDA software and was not confounded by usability issues that would arise due to a lack of understanding and grasp of the desktop, mouse and keyboard setup and the Windows, Icons, Menus and Pointer (WIMP) interaction paradigm. Moreover, the profile and technology skillset of older adult users is rapidly changing [44]. It is therefore important that present-day studies do not misrepresent the needs of near-future older adult populations who will be the users of emerging technologies and applications [45]. It has been suggested that carrying out task-focused usability sessions with older adults who possess basic computer literacy skills is a useful strategy for eliciting insights into the needs of near-future older adult user populations, who are more likely to possess basic computer literacy skills as a consequence of being more exposed to a range of technologies throughout their life-course [44–47]. The number of participants recruited for this study is in excess of the recommended threshold of five participants typically considered as a good starting point to carry out effective think-aloud interaction and usability testing studies [48, 49]. Six of the participants were female and five were male. All participants were retired with the exception of one who was employed part-time and another who was employed full-time. The mean age of participants was 68 years of age with a standard deviation of 5.70. Two participants were recruited by word of mouth and eight of participants were recruited from the ‘Active 50’s’ exercise group at Brunel University Gym. The ‘Active 50’s’ group consists of approximately 120 members, who are aged 50 years and over. No financial incentives were offered to take part in the study hence participation took place purely on a voluntary basis. Table 1 provides a summary of participant profiles for this study.

**Table 1:** Summary of community dwelling older adult profiles

Participant	Gender	Age	Occupation
A	Male	66–70	Software developer
B	Female	56–60	Teacher
C	Female	66–70	Personal assistant
D	Male	76–80	Engineer
E	Male	66–70	Market researcher
F	Male	71–75	Administrator
G	Male	61–65	Financial adviser
H	Female	66–70	NHS Manager
I	Female	66–70	Teacher
J	Female	66–70	Analyst

### CIDA interactive usability sessions

Task-focused interactive usability sessions lasted for no longer than 90 min in total for each participant. There were four key stages to each session which were as follows:

1. Issue information sheet, answering of questions, completion of informed consent form.
2. Demonstration of software application and question and answer session.
3. Task-focused design task using the custom CIDA software application.
4. Interview about perceptions/experience of using the software application.

On arrival, information sheets were distributed to users prior to participation in the session, the content of which was worked through with each participant individually. The information sheet provided a brief background and context and purpose to the study, and summarised the main activities that would take place during the course of the session. Participants were encouraged to ask questions throughout the process, and any questions were answered as they arose. Participants were then asked to complete a consent form in which their ethical rights were explained in terms of informed consent, withdrawal and anonymity.

**Fig. 2:**
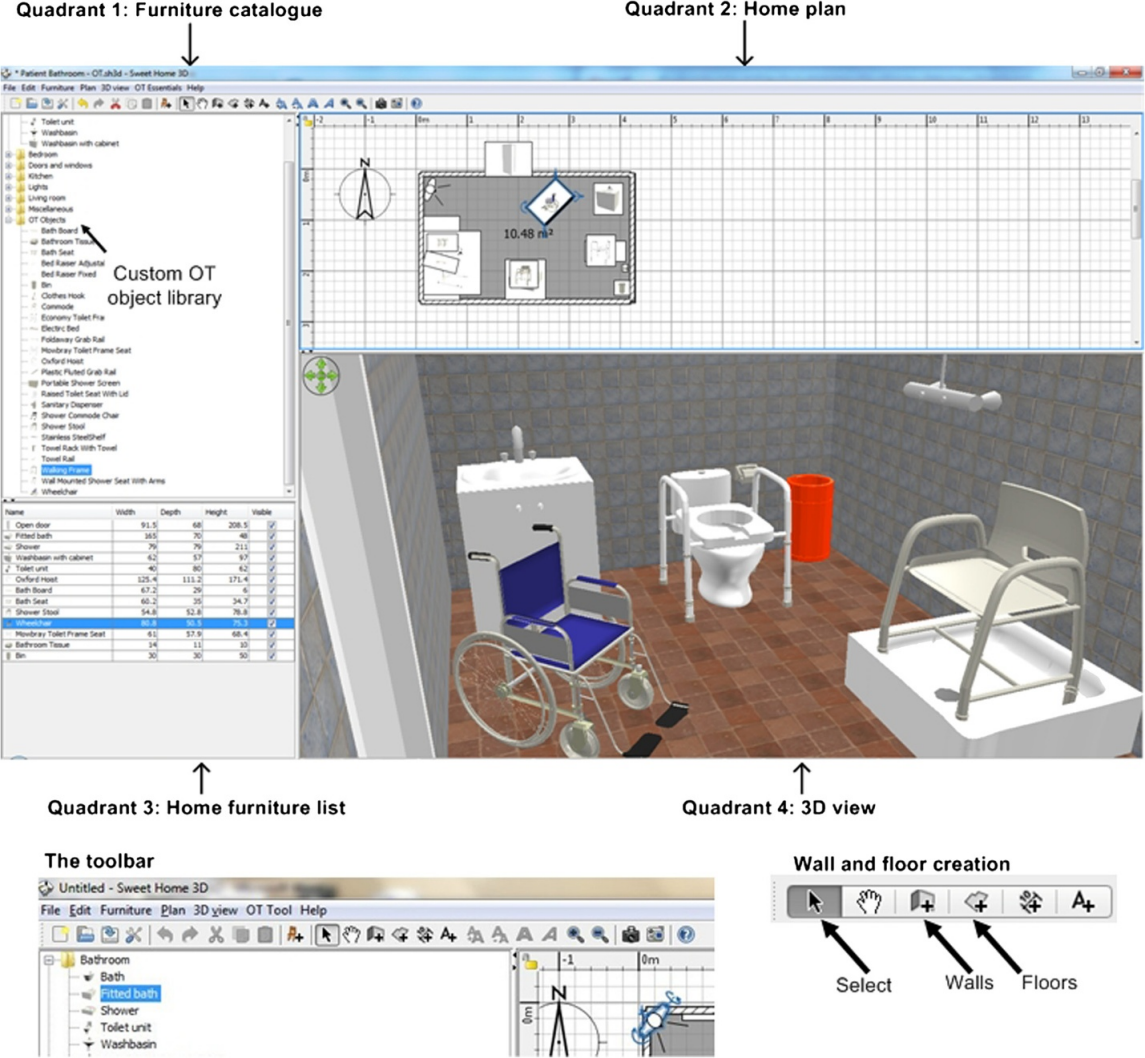
The SweetHome 3D application interface

Participants were then given a 10 min demonstration of the SweetHome 3D application. As part of this presentation, participants were shown how to carry out key tasks such as creating a floor plan, walls and inserting items of furniture into the 3D environment. The CIDA software application used for the purposes of this task was a customised version of SweetHome 3D [50]. Figure 2 illustrates the customised SweetHome 3D application interface which was presented to the participants and which was used to design and develop home interiors by participants in each session.

The application interface for SweetHome 3D consists of four functional quadrants: 1) Furniture catalogue; 2) Home plan, 3) Home furniture list, 4) 3D view. The application used in this study has been customised to include a library of OT specialist assistive equipment necessary for OTs to make typical home adaptation recommendations as part of the pre-discharge home visits process. The 3D models of the assistive equipment were all custom built for the purposes of this research, and were developed using Blender, an open sourced 3D modeling application [51]. These artefacts were presented within the furniture catalogue quadrant of the application within a folder entitled “OT Objects”. Occupational therapy assistive devices featured in the library included a bath board, a shower chair, sanitary dispenser, portable shower screen, shower commode, bath seat, bath hoist, ramps, a range of grab rails, a wheelchair, raised toilet seat and toilet frame. The custom OT objects library folder and how this was integrated into the furniture catalogue navigation pane and examples of some of these OT objects are also presented in Fig. 2 (Quadrant 1: Furniture catalogue) as well as how these OT objects may be modelled within an example 3D view of a bathroom environment (Quadrant 4: 3D view).

After the demonstration of the software and a question and answer session, participants were provided with basic written instructions, presented in Table 2, outlining the key steps necessary to complete the main interactive session task.

**Table 2:** Written instructions for interaction task with SweetHome 3D

*Participant Instruction Sheet*
1. Create a room with four walls, one door and one window and a floor.
2. Add a bathtub, followed by a bath board to the room (the bath board is to sit across the bath width ways).
3. Add a toilet, followed by a toilet frame to the room (the toilet frame is to sit around the toilet).
4. Finally, add a shower, followed by a shower chair to the room (the shower chair is to sit inside the shower tray).

Note: Dimensions and location of items within the room are entirely your choice

For the main task, participants were asked to design a bathroom of their choice. As part of the task, they were asked to insert assistive equipment i.e., a bath board, toilet frame and shower chair in positions they deemed appropriate. Participants were asked to design the bathroom from scratch, whilst adopting a ‘think aloud’ approach, which enabled them to verbally share their thoughts whilst interacting with the application [52]. The think-aloud technique is a well established technique used for eliciting real-time thoughts of users as they interact with software applications. The technique is particularly useful as it elicits user preferences but also provides valuable insights into the reasoning behind these preferences and thoughts. It has been used extensively within the context of usability testing, and with regards to its use within occupational therapy, it has been used to explore clinical reasoning [53]. Users were reminded throughout the task, that there was no urgency in them completing the task, and they were encouraged to take as long as they required to provide comments and to interact with the application. Standard think-aloud prompts such as “what are you thinking?” and “what are you doing now?” were used whenever the researcher felt there were extended periods of silence [54]. Written notes were taken throughout each session and all sessions were also audio recorded. A short interview was carried out with each participant at the end of the interactive usability task. Participants were asked to reflect on their experience of using the application and discuss the barriers and opportunities of using a CIDA application as an assistive tool and any usability issues they experienced during the session.

### Data analysis

Template analysis refers to the process of organising and analysing qualitative data [55]. This involves developing a template of codes that reflect the themes of importance that emerge from the dataset. The analysis approach taken to the dataset was both inductive, as some sub-themes were closely linked to the data, and deductive as the high-level themes were driven by theory and the researchers’ analytical interest [56]. The first stage involved creating a template which used the pre-defined codes specified by the Technology Acceptance Model (TAM). Hence, analysis considered the participant perceptions of the CIDA application in the context of the three high-level TAM themes relating to the Perceived Usefulness (PU), Perceived Ease of Use (PEOU) and Actual Use (AU). Carrying out the analysis in this way conforms to what is considered to be a contextual constructivist approach to thematic analysis [57]. Initially, all interactive usability sessions and associated interview recordings were transcribed verbatim. The entire dataset was then read and comments were assigned to the three pre-determined TAM themes and moving similar texts into one place and re-reading segments to ensure that connections were justified [58]. The dataset was then examined iteratively through several stages of splicing, linking, deleting and reassigning sub-themes within each pre-determined high-level theme. Sub-themes in the context of individual participants’ accounts were considered, as well as examining the data across participants. Sub-themes were included because of their relevance to the research question and not necessarily because of their prevalence across the data set, as is acceptable in qualitative research [56].

### Ethical considerations

The study was reviewed and approved by the Brunel University Research Ethics Committee prior to any data collection. All participants taking part in the study were guaranteed confidentiality and anonymity. Signed consent forms were obtained from all participants prior to taking part in the semi-structured interviews. Participants were informed of their right to withdraw from the study at any time. This was done both in writing and verbally.

## Results

**Fig. 3:**
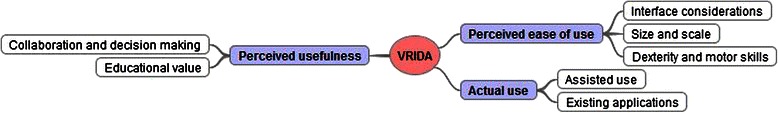
Thematic mind map of themes and sub-themes

In this section, the results of the task oriented usability sessions and associated interviews are presented. Primarily, results are presented within the three key TAM themes used for analysis: Perceived usefulness; Perceived ease of use; Actual use of the technology as well as the associated sub-themes that were identified within these high-level thematic constructs. Figure 3 presents a summary of the themes and sub-themes in the form of a thematic mind-map.

### Perceived usefulness

#### Collaboration and decision making

Participants felt that the CIDA software provided clear and useful 3D visualisations of the home environment. They commented that these 3D home representations were understandable and clearly communicated their ideas about the home layout. Participants commented that CIDA could serve as a valuable tool for joint decision making and collaborating with others in order to discuss and explore possible ideas for adaptations to the home environment. They also noted that it could enable the patient to have more influence on the decisions that are to be made about adaptations to their home environment whilst also providing a clear visual representation to the patient of the potential changes that may be proposed by the practitioner.

*Participant J: “This works well, you can look at the different options together and explain better what you mean, I mean also understand what they mean, yes really like the idea of it…”*

Some participants also suggested some useful scenarios in which they felt that the software could be a useful tool to help the OT to collaborate with a client and illustrate to them the types of changes that could be made to their home. But also importantly, a tool which would empower the patient to better voice their preferences but also increase their awareness and understanding of the feasibility of a variety of options that may be available to them. Furthermore, it was felt that the patient would have a more realistic idea of the implications of proposed changes, and how these changes could potentially impact upon their day-to-day functioning within an adapted environment.

*Participant B: “Yes this is excellent, and of course, you know as a little lady you get ‘oh no I don’t want that’, but if you physically see it put in, it’s excellent, and I can physically see that that couldn’t fit in there and, or because my toilet’s fitted I couldn’t use, you know, this that and the other”*

One of the challenges in the process of making decisions about home adaptations arises when the OT is proposing potential adaptations to the patient. Indeed, it was envisaged that the CIDA software could actually enable OTs to better communicate to patients the feasibility of proposed solutions and enable the patient to perhaps better understand and embrace changes that otherwise may have seemed not possible or may have been ruled out in the absence of a 3D representation of the possible changes.

*Participant B “… if you were physically drawing it [using the SweetHome 3D, you can say, ‘oh get rid of that bath love’ and put a shower in and you’ll have so much more space, and they can physically see that that would be really good … because a lot of old people say no that’s impossible, move on. Whereas if you physically drag, got rid of the bath and put a shower unit there, they say, ‘oh there’s space around the toilet now, yes that looks alright’”*

Interestingly, some participants reported being already familiar with the use of CIDA software, and have found it very useful when used it in collaboration with interior designers to negotiate home improvements with high-street vendors. The CIDA software was reported to typically being used as a collaborative tool, as opposed to a tool which the participant would use on their own. Typically, an expert user would draw up plans of a proposed interior design, which then would be used as a mock-up around which a discussion could be had. The prior experience of using such software, however, was seen as positive and served as a useful tool which facilitated enhanced shared understanding and improved joint decision making.

*Participant F: “It’s hard sometimes to know what’s meant when someone says, move this here, take this out and put a sink in there. At least with this, I can actually understand what they mean and see if it’s something I want… I had exactly this conversation with the man from [a high-street DIY store] when we had our new kitchen designed and fitted”*

#### Educational value

Perhaps unsurprisingly, the majority of participants were unfamiliar with the occupational therapy objects that were included in the customised furniture library of this application. The participants were asked to incorporate a minimum of three specialist ‘OT objects’ into their plan, including a bath board, a shower stool and a toilet frame. Out of the ten participants, two were familiar with the equipment, one a retired physiotherapist and the other, her partner.

*Participant E: “What’s a Mowbray Toilet Frame, sorry I don’t know these terms”*

A lack of knowledge of the OT specialist objects impacted upon the perceived usefulness of the application. It was difficult for participants to know whether they were actually proposing solutions which were of any real value to them in reality and meant that participants were unclear of the function of the specialist objects and how the equipment should be fitted. They were unclear how the person, environment and equipment interacted within a real life setting, something that an occupational therapist may take for granted. As a result they did not always make safe suggestions for placing the equipment in the room.

*Participant C: “So this presumably is either something that you’d sit on and it would go down into the water or it’s […] to step on” Participant C*

*Facilitator: “A bath board is something that sits across the top of the bath”*

It was clear that the intended function of specialist OT objects was not intuitively understood by the lay user, despite being presented with its name and 3D visual representation of the object. Only those participants with prior knowledge of such items were able to independently make decisions about placement of these objects within their home environment. However, as a result of discussing the function of OT objects with the facilitator, participants quickly developed their understanding of the intended function of specialist equipment.

*Participant A: “Right, I don’t know what the framework is for, that goes round it”*

*Facilitator: “… It’s a piece of OT equipment, the toilet frame … helps you lever yourself on and off.”*

At the end of the interactive sessions participants successfully proposed the use of specialist OT objects within their home environment by the end of each session. The interactions between the facilitator and the participants, therefore, did highlight the educational value that could be realised as a result of having a conversation around the function of the OT objects whilst using the CIDA application to model these objects within the 3D home environment.

### Perceived ease of use

There were a number of issues identified, in terms of the usability of the software, by this sample cohort. Bringing to light that in its current form, it is unlikely that CIDA application could be used independently by patients within the home setting in order to propose adaptations to practitioners.

#### Interface design considerations

Participants found it challenging to interact with numerous aspects of the CIDA user interface. For example, establishing the function of various buttons in the menu bar at the top of the quadrant view was a theme which reoccurred with a number of participants. Participant B noted that the small icons meant the software was not *“user friendly”.* In particular, this was considered to be as a result of the application not being designed specifically for use by older adults and consequently, and the size of visual icons representing the function of each button being too small.

*Participant A: “these icons are quite small … if it was bigger you might have a better chance of seeing [them]”.*

In order to manipulate the size and position of furniture objects which had been placed into the ‘Home plan’ and ‘3D view’ quadrants, participants were expected to hover the mouse pointer over respective furniture objects before selecting them. Participants reported that they struggled to click on or hover over objects of furniture largely due to their size.

*Participant C: “I think it’s so intricate to actually get that really right change in the size of an object I would have a lot of trouble”*

Visual sign-posting and textual descriptors of the function of the buttons on the menu bar was also suggested as a requirement if the interface was to be more usable for participants. The visual representations used to communicate the function of each respective button was not considered to be sufficient for the needs of the user group.

*Participant G: “It’s knowing what the controls actually mean […] I mean there are no signposts to tell you”[…] it would just be useful to know that on the controls, on the corner, on the object itself, this corner means this, that corner means that”.*

Difficulties were also noted in terms of finding appropriate objects of furniture and specialist OT equipment within the furniture library. Identifying appropriate objects and placing these into the Home plan was a core aspect of the task, however, one which also caused difficulties. All of the items were located in folders, according to the room they would most often be found in, on the left hand side of the screen. However, there was a separate folder for customised ‘OT objects’. It was suggested that perhaps the OT objects should be placed within the respective room folders as opposed to being placed in a separate folder. This would help users know which room the OT objects could be used in. One participant noted that it was time consuming to find the objects she wished to place in her plan.

*Participant C: “It was quite complicated … and I would say I’m reasonably computer literate, but there was a lot involved, up and down to find the bits I need … if I’m totally honest I would have given up [on my own] … I’m not an occupational therapist, but you’ve got 8 gadgets used in the bathroom … instead of this whole list is to have physical pictures of those 8 items and slot them in [to the bathroom folder]. ….*

#### Size and scale

The notion that developing home interior representations tended to be time consuming was compounded by the fact that many participants felt it was important to have accurate measurements of furniture objects and floor plans of the rooms being modelled. This outcome was unexpected, particularly when considering that participants were given clear instructions that dimensions and location of items within the VR models were to be decided upon at the discretion of the participant. Nevertheless, participants spent a considerable amount of time exploring whether the scale of the floor plan looked realistic and indeed whether the size of the furniture items seemed reasonable in the context of the floor plan they had created. This seemed important to participants who believed that in the absence of correct scale and size, the exercise would have little value to either party.

*Participant I: “On scale, the room has to be set out … all the measurements of the bathroom need to be to scale.”*

Some participants felt that it would be useful to be provided with the floor plan of the house/flat prior to starting the task of populating it with furniture and customising it to represent their own personal space. It appeared to be difficulty for participants to propose approximate measurements of floor plans and represent these within the application without any guidance on this matter.

*Participant C: “You’d need to have the proportions of actually what you are going to, what size the flat is actually going to be. This is the size the bathrooms going to be and then you can say right, if that’s the size it’s going to be this is the best place to put the different things…”*

#### Dexterity and motor skills

The skills required by the individual to use the software were highlighted throughout the sessions, both by the participants themselves and through an observation of their actions. Good eyesight was noted to be essential to see the items on the screen, particularly due to their size.

*Participant H: “You’ve got to be quite dexterous especially when you’ve got a small object”.*

It was also noted that dexterity and fine motor skills were required to use the software successfully. Participants noted the size of the objects required her to be quite skilful to position them on the plan

*Participant H: “Because if you’ve got the bath close to the wall and you’ve got to turn it … You actually end up moving the wall, which you don’t want to do, by moving the object or the bath. The bath board. It’s quite delicate.”*

*Participant B: “I think somebody, not necessarily with worse eyesight than me but that might have problems with their fingers … [it] might be a problem”.*

### Actual use

#### Assisted use

Perhaps as a consequence of the significant challenges that participants faced when utilising the CIDA software, in general participants did not believe that patients could use this application on their own, in its current form. When discussing the potential actual use of this application in practice, participants generally believed that users would need to be supported by an expert user, perhaps an occupational therapist, which could explain the function of specialist OT equipment and also assist in using the application. Typically, participants believed that considered the level of support people would require if the software were used in reality, between an occupational therapist and a client.

*Participant B: “Personally I don’t think the little old lady would want to do the actual putting in […] I think she’d probably want to tell you […] the baths here, that’s here, that’s there”.*

*Participant A: “I think you’re going to rely on an awful lot of knowledge … and you know, I think they’re going to need someone like you [Occupational Therapist] sitting with them…but that’s not unreasonable”.*

Participants were however positive about the prospect of actually being involved in the use of this software alongside practitioners. It seemed that the potential for becoming a participant in the decision making process and working alongside practitioners as joint experts is something that some participants believed would be a valuable opportunity which they looked forward to being part of.

*Participant F: “… it’s a case of, here use this, or take this medicine without explaining why. Using this [software application] with someone like a therapist would be much better, I’d love to do that, yes, I think everyone would like to do it …”*

In terms of the scenarios in which this software was perceived to be useful, however, some participants felt that it may only be useful for major adaptations to patient’s homes or indeed planning new-builds for particular patient groups as opposed to being used for minor adaptations. This was the case, particularly in light of the considerable overhead that participants saw in using the application to develop an initial floor plan to scale and then populate it with basic items of furniture.

*Participant J: “I wouldn’t want to have to draw up a whole plan of my bathroom and then end up only putting in one toilet frame afterwards....”*

*Participant H: “I think it would be very useful for actually, if you are going to be designing disabled flats for disabled people”.*

#### Existing applications

An interesting finding was that a number of participants already had prior knowledge of CIDA software and discussed examples of where they had personally had some knowledge/experience of its use in practice. This appeared to impact positively on their views of the potential feasibility of using such software applications within an OT context. In general, participants were positive about it’s potential utility in practice and used case examples to substantiate their view of it being of potential value within the OT context.

*Participant G: “… it is excellent, because a lot of people can’t visualise things, so, even if you go to Wickes for a kitchen or something, they will do this and it is very, very useful”*

*Participant C: “Oh it’s very clever and I find this interesting because I know estate agents use it”.*

Interestingly, when discussing the actual contexts in which CIDA could be deployed, one participant reported having used similar home design software in the past when designing their own rooms at home.

*Participant D: “I did mine when we had our extension, I designed the kitchen for the extension we had, I worked everything, where I wanted it to go, how big a window I wanted”.*

## Discussion

With the increased need to deliver person-centred care, coupled with the recognised growing potential use of technology within the healthcare, there is a need to identify new and innovative uses of technology which respond to these needs and exploit the functionality of emerging technological innovations within the healthcare context. This study considered community dwelling older adults’ perceptions of using a customised virtual reality interior design application as a tool to assist and engage patients in the pre-discharge home visits process. A total of ten participants used a CIDA as part of a home interior design task which incorporated the use of specialist occupational therapy assistive equipment. A think aloud perceptions and semi-structured interviews were carried out with participants during and after the interactive design task. Analysis of think aloud and interview data aimed to gain detailed insights into the key technology acceptance model (TAM) factors: perceived usefulness (PU); perceived ease of use (PEOU); and actual use (AU) of CIDAs as part of the pre-discharge home visits process. The results revealed a number of sub-themes that related to the TAM factors. Table 3 provides a summary of the themes, sub-themes and the associated study outcomes.

**Table 3:** Study outcomes

Theme	Sub-theme	Study outcomes
PU	Collaboration & decision making	• Clear and understandable 3D home representations
		• Valuable tool for joint decision making and collaboration
		• Empower patients to have influence on decisions
		• Increase awareness and understanding of the design options
	Educational value	• Function of specialist OT equipment not intuitive
		• Potential of learning equipment function from using CIDA
PEOU	Interface considerations	• Increase size of icons
		• Provide textual descriptors
		• Revise folder structure/location of OT equipment
	Size & scale	• Accuracy of measurements
	Dexterity & motor skills	• Redesign interface/functions to require less dexterity/motor skills
AU	Assisted use	• More suitable to be used alongside an expert user/practitioner
	Existing applications	• Confidence in value and applicability
		• Familiarity with CIDA in existing real-world contexts

Based on the findings of this study, in terms of *PU*, patients see the use of CIDA as a promising solution which they believed generated intuitive and understandable representations of the home environment. Importantly, participants expressed the view that CIDAs would serve as a valuable tool to facilitate patient/practitioner collaboration. Furthermore, CIDAs were seen as being a potentially important and useful visual aid which would facilitate shared understanding of the purpose and function of the proposed home adaptations. This is particularly valuable given that, to date, insufficient explanation and notification of home adaptations during home visits has resulted in some users feeling dissatisfied with their experience resulting in equipment abandonment levels in excess of 50 % [12]. Enabling people to stay at home and maintain independence at home can add to an increased sense of control and improved quality of life [59–61]. Interestingly evidence from a study involving older adults from eleven European countries found that older adults wanted to have a trusting relationship with the practitioners, to be respected about their preferences, and to receive clear health information from the healthcare providers [62]. CIDAs were seen as having potential to help facilitate the delivery of healthcare according to all of these factors and to improve patient/practitioner communication and collaboration within the pre-discharge home visits process.

It was also noted that the use of CIDAs would help to enable patients to be more involved in decisions made about their care, hence improving the potential for shared decision making about home adaptations, reducing anxiety, and empowering patients to become more equal partners within the pre-discharge home visits process. A key enabling factor for patients to be empowered is the promotion of health literacy. Health literacy is defined as the ability to ‘access, understand, evaluate, and communicate information as a way to promote, maintain, and improve health in various settings over the life-course.’ [63]. Traditionally health literacy tools have typically taken the form text-based information leaflets [64]. However, a strong relationship exists between poor literacy skills and poor health outcomes [65]. Therefore, the use of more visually focused health communication tools such as CIDAs, are likely to provide the opportunity to overcome some of the communication imbalances that exist in current practice given the current reliance on health leaflets which are not effective for patients with poor literacy. A recent study exploring the use of a virtual reality application to assess whether it could be used for persons with intellectual disabilities to achieve improved levels of health literacy has achieved very promising results [66].

In terms of *PEOU*, despite all participants self- reporting to be computer literate, the current application interface requires further development if it is to be considered usable by community dwelling older adult patients. There were a number of usability issues suggesting that the size of the icons were too small, that textual descriptors describing the function of buttons and icons were missing and the folder locations of furniture within the application were not intuitive. Furthermore, the current design of the user interface appears to be overly demanding in terms of the motor skills and level of dexterity required from the user which seems to impact upon the extent to which participants believed they would be able to use the application independently. Indeed existing research relating to interface design for older adult users suggests that many of these usability issues may be overcome if design considerations specific to this user group are adhered to when designing and developing the user interface and system functionality [61, 67].

There was also a tendency for older adults to expend significant effort to ensure that the measurements in terms of size and scale accurately represented the real-life equivalent of the home environment being modelled, despite being advised that this was not necessary for the purposes of the set task. The personal home reflects notions of identity, expression of self, and a sense of control [68]. Indeed older adults have reported appreciating the independence of living at home and the autonomy that living in their own home affords them [69]. Evidence suggests that older adults have an increased reluctance to leave home, and a deeper emotional attachment to their home than their younger counterparts (Saunders 1989) even to the extent that they would stay in their home when, as a physical home, it may be less suitable [70]. This is a potentially time consuming overhead which could present a challenge if the CIDA was used independently by patients.

From the perspective of *AU*, participants expressed a preference for using the application in an assisted context, i.e., alongside a practitioner, as opposed to independently. This preference may in part be as a result of the interface design considerations which were noted as a function of PEOU and hence potential users may feel more confident to use the application independently if the application is developed further to overcome some of the usability issues noted. Nevertheless, the community dwelling older adult participants also suggested that the use of CIDAs offered the valuable potential of discussing ‘different options together’ which, regardless of the usability of the application, would deliver a valuable opportunity for patient/practitioner interaction and shared decision making which currently is lacking in the pre-discharge home visits process [9, 12, 71]. The potential applicability of the CIDA within real-world settings, had clear potential according to participants, some of whom reported to have had personal experience of using similar software applications in more commercial contexts, for example when redesigning their home kitchen. This is a promising finding which is likely to support the potential actual use and adoption of CIDAs as a useful assistive tool within the occupational therapy setting and the pre-discharge home visits process.

When comparing the findings of this study with those of the study exploring the use of CIDA with occupational therapists [26], It is clear that there are some similarities and differences. Both older adults and occupational therapists perceived the use of CIDA as a promising solution which they believed generated intuitive and understandable representations of the home environment. Importantly, participant groups in both studies expressed the view that CIDAs would serve as a valuable tool to facilitate patient/practitioner collaboration, communication and empower older adults to be more involved in decisions about their care. This could be a positive feature of CIDA, compared with other communication tools/devices used in this space, such as information leaflets. Effective information exchange between older adults and health care professionals is essential to ensure active participation in decision making and maintain older adults sense of self-worth and dignity [72]. This is a particularly important factor when considering that home visits can have a negative impact upon care if decisions are not made collaboratively and if older adults and therapists have different perceptions [9]. In addition older adults who are involved in a meaningful way in decisions surrounding devices are likely to be more satisfied and less likely to abandon using specialist devices and installed items of equipment [73]. Communication is critical for providing efficient and effective care to patients. However, there were noticeable differences between the two groups with regards to how they believed the CIDA could actually be used in practice to improve communication between patients and practitioners. Whilst both occupational therapists and most older adults were able to create 3D representations of their homes, service users expressed a preference for using the application in an assisted context, i.e., alongside a practitioner, as opposed to independently. This suggests that older adults may value the expertise and knowledge of health care professionals and value their input within the communication process as suggested, existing research support this finding [71]. In addition, working together in a more collaborative way could help to promote more positive practitioner attitudes towards older adults, and enhance trust between practitioners and older adults. Research has found that family members may find communicating with professionals and contributing to care decisions as challenging [74]. The use of CIDA in an assited context, as suggested by community-dwelling older adults in this study, may provide valuable opportunities to begin to overcome some of the challenges that patients and family members experience when contributing to care decisions.

### Study limitations

The participants who took part in this study all self-reported to be healthy, active and familiar with the use of desktop computers. It is therefore important to note that this sample may not be representative of the typical groups of older adults that OTs frequently engage with, and should be taken into consideration when interpreting the results. Indeed, it is likely that OTs and other health professionals frequently deliver care to older adults who have, for example, serious co-morbidities, difficulties with vision and memory, and some level of cognitive impairment and dementia. With regards to the typical level of ICT experience possessed, however, the typical older adult patient profile is changing, as younger and more technologically aware generations make the transition into the older adult category, so the typical level familiarity with ICT of this cohort will increase [44]. Therefore, although the sample in this study is biased, such participants were recruited with the motivation of gaining insights from a sample, that may to some extent, better represent the more technologically aware older adult user group of the future. The number of participants that took part in this study may be considered to be too small to make generalisations about older adult perceptions of the use of CIDAs in the pre-discharge home visits process more generally. Furthermore, a limitation of the qualitative data collected for this study meant that quantifiable outcomes in terms of the effect of CIDA as an intervention before and after hospital discharge, and its comparison against a control group, was not explored. This direction of enquiry would have provided further tangible insight into the clinical effectiveness of using CIDA within the pre-discharge home adaptations process and should be considered in future, once the software application has been further developed to incorporate the end-user needs revealed in this study. With regards to sample size, however, the number of participants that took part in this study exceeds the minimum starting point of five participants that are necessary to provide useful and effective feedback when using the think-aloud protocol for research with interactive software applications [48]. The deductive approach adopted in the initial phase of analysis, via the use of the three high-level TAM themes, may also be considered to be a limitation of this study. In particular, this approach may have reduced the breadth of themes that may have emerged from the data if a purely inductive approach was adopted. However, adopting a deductive approach also afforded the analysis to focus in more detail on factors that related specifically to technology acceptance, which was the aim of this study.

## Conclusions

In summary, we have investigated from a TAM perspective, community dwelling older adults’ perceptions of using a customised CIDA as an assistive tool in the pre-discharge home visits process. As a result of completing a home interior design task using the tool, valuable insights were gained into the potential value and utility of using a CIDA within the occupational therapy setting and more specifically within the pre-discharge home visits process. Notably, participants found the 3D interior models of the home clear and understandable, they saw clear potential for using the tool to facilitate joint decision making about home adaptations and to improve their understanding of the potential value and function of proposed adaptations. Community dwelling older adults suggested the usage scenario which would most likely be of value, would involve both the patient and the practitioner using the customised CIDA application collaboratively in a face-to-face setting. Further research is needed to explore patient and practitioner perceptions of using CIDAs collaboratively as part of the pre-discharge home visits process. Additional development work is also needed to incorporate the requirements suggested by participants as a result of this study and to identify practitioner specific requirements which will ensure that both patients and practitioners are able to optimally benefit from using this application in practice.

## Abbreviations

3DVE: Three-Dimensional Virtual Environment
AU: Actual Use
CIDA: Computerised 3D Interior Design Applications
OT: Occupational Therapist
PEOU: Perceived Ease of Use
PU: Perceived Usefulness
TAM: Technology Acceptance Model
